# The Impacts of Different Air Pollutants on Domestic and Inbound Tourism in China

**DOI:** 10.3390/ijerph16245127

**Published:** 2019-12-15

**Authors:** Xiaowei Xu, Daxin Dong, Yilun Wang, Shiying Wang

**Affiliations:** School of Business Administration, Southwestern University of Finance and Economics, Chengdu 611130, China; xiaoweix@swufe.edu.cn (X.X.); wangyilun@smail.swufe.edu.cn (Y.W.); sherringw@smail.swufe.edu.cn (S.W.)

**Keywords:** air pollution, PM_2.5_, PM_10_, SO_2_, NO_2_, tourist arrivals

## Abstract

Previous studies have reported that air pollution negatively affects the tourism industry. This paper attempted to answer the following question: among different air pollutants, which one acts as the most adverse factor? The study was based on a sample of panel data covering 337 Chinese cities for the period between 2007 and 2016. Four pollutant indicators were inspected: PM${}_{2.5}$ (particulate matter 2.5 micrometers or less in size), PM${}_{10}$ (particulate matter 10 micrometers or less in size), SO${}_{2}$ (sulfur dioxide), and NO${}_{2}$ (nitrogen dioxide). It was found that PM${}_{2.5}$ had a significantly negative impact on both domestic and inbound tourist arrivals. Regarding the other three pollutant indicators, except for the negative influence of NO${}_{2}$ on inbound tourist arrivals, no statistically significant impact was found. This study suggests that tourism policy makers should primarily focus on PM${}_{2.5}$, when considering the nexus between air quality and tourism development. According to our estimates, the negative impact of PM${}_{2.5}$ on tourism is substantial. If the PM${}_{2.5}$ concentration in the ambient air increases by 1 μg/m${}^{3}$ (= 0.001 mg/m${}^{3}$), domestic and inbound tourist arrivals will decline by 0.482% and 1.227%, respectively. These numbers imply an average reduction of 81,855 person-times in annual domestic tourist arrivals and 12,269 in inbound tourist arrivals in each city.

## 1. Introduction

In recent years, the tremendous industrial growth of the Chinese economy has caused high levels of air pollution in some regions. Air pollution heavily affects public health. For instance, it was reported that air pollution has caused an average of 1.1 million premature deaths in China annually [1]. Moreover, air pollution also inhibits many economic and social activities. In particular, the adverse effect of air pollution on tourism has received increasing attention. Pollution damages tourism’s development by evoking negative psychological states in tourists, reducing the aesthetics of scenic spots, harming the tourist experience, and decreasing tourism’s demand (e.g., [2,3,4]).

Prior studies have found that air pollution negatively influences tourism’s development and activities. The air pollutants examined included PM (particulate matter), SO${}_{2}$ (sulfur dioxide), NO${}_{2}$ (nitrogen dioxide), and so on. Among them, PM is one of the most well-known types of air pollutants. PM with a diameter of 2.5 micrometers or less is known as PM${}_{2.5}$, while that with a diameter of 10 micrometers or less is known as PM${}_{10}$. At present, given that social media and news agencies frequently associate PM${}_{2.5}$ with haze pollution, many people perceive PM${}_{2.5}$ and haze weather to be interchangeable concepts [5]. Additionally, some people regard PM${}_{2.5}$ as the only pollutant necessary for measuring the air quality index (AQI). Although PM${}_{2.5}$ is a dominant pollutant in haze pollution, it should be noted that, according to the World Health Organization (WHO) Air Quality Guidelines, relevant pollutants also include PM${}_{10}$, NO${}_{2}$, SO${}_{2}$, and ozone (O${}_{3}$). Different air pollutants have been used together to calculate AQI scores and have also been found to be associated with negative health outcomes, such as hospital admissions, respiratory diseases, incidence of asthma symptoms, and cardiovascular disease (e.g., [6,7]). When SO${}_{2}$ and NO${}_{2}$ combine with water and sunlight, the main component of acid rain results, which can cause deforestation and destroy cultural heritage, such as ancient historical buildings and monuments.

Yan et al. [8] empirically examined the effects of different air pollutants on urban activities in China using geotagged check-in records on a Chinese social media platform, indicating that SO${}_{2}$ had the largest impact, followed by PM${}_{2.5}$, NO${}_{2}$, and PM${}_{10}$. They further discovered that leisure-related activities were much more sensitive to air pollution than work-related activities. To examine the impact of air pollution on the tourism industry, a number of studies have used PM${}_{2.5}$, PM${}_{10}$, or AQI as indicative measures of air quality (e.g., [9,10,11,12]). However, how other major air pollutants (e.g., NO${}_{2}$, SO${}_{2}$) influence the tourism industry in China has seldom been explored. Given that these air pollutants could all pose health threats to travelers [13] and destroy the attractiveness of destination cities to potential tourists, knowledge about how and to what extent major air pollutants exert impacts on tourism industry is required.

To address the above literature gap, this study aimed to examine the impact of air pollution on the tourism industry by taking into account four major air pollutants: PM${}_{2.5}$, PM${}_{10}$, NO${}_{2}$, and SO${}_{2}$. More specifically, this study examined whether and to what extent the different air pollutants respectively impact domestic and inbound tourism. The study’s results are expected to help the Chinese government formulate better air quality control strategies, in order to maintain a sustainable tourism industry. Additionally, the results of this study could help the public health sector better understand how to issue travel advice on air pollution.

The rest of this paper proceeds as follows. Section 2 presents a literature review. Section 3 discusses the empirical model and the data used in the analyses. The estimated results of the empirical model are reported in Section 4. Section 5 discusses the implications of the results. Section 6 concludes and talks about the directions for future research.

## 2. Literature Review

### 2.1. Air Pollutants: Sources and Impacts

SO${}_{2}$ and NO${}_{2}$ are among the major causes of smog and acid rain. SO${}_{2}$ arises from industrial activities that burn fossil fuels (e.g., coal, oil, and diesel) containing sulfur. Sources include but are not limited to power plants, metal processing and smelting facilities, and diesel vehicles and equipment [14,15]. Common effects of SO${}_{2}$ are respiratory problems and increased hospital admissions for cardiac disease [16]. NO${}_{2}$ is typically produced from combustion processes (e.g., heating, power generation, and engines in vehicles and ships). NO${}_{2}$ emissions are more likely to be clustered in densely populated urban areas and suburban industrial areas [17]. High levels of NO${}_{2}$ exposure could cause respiratory infections and the prevalence of bronchitic symptoms in asthmatic children aged between 5 and 14 years old [6]. NO${}_{2}$ exposure has also been found to be associated with lung cancer [18], mortality, hospital admissions, and respiratory diseases across all ages [6]. In addition to the health effects of SO${}_{2}$ and NO${}_{2}$, their environmental effects are largely due to the acid rain that forms from SO${}_{2}$ and NO${}_{2}$. It is well known that acid rain not only damages natural ecosystems, but also man-made materials, such as limestone, marble, and sandstone [19]. For example, the Giant Buddha at Leshan in Sichuan Province, the Longmen Grottoes in Henan Province, and the Dazu rock carvings in Chongqing, which are famous tourist attractions in China, have been reported to be at a high risk of rapid deterioration from acid rain [20].

Compared to SO${}_{2}$ and NO${}_{2}$, PM is more tangible and visible. Wang et al. [21] identified soil dust, vehicular emission, coal combustion, secondary aerosol, industrial emission, and biomass burning as six common sources of PM${}_{2.5}$ and PM${}_{10}$ in Beijing, China. A high concentration of PM directly reduces the visibility of air. It is also well known that PM severely damages public health [22,23,24]. For the Chinese population, Lu et al. [25] found that short exposures to PM${}_{2.5}$ and PM${}_{10}$ were positively associated with increases in mortality due to cardiovascular and respiratory disease. Feng et al. [26] further suggested a strong association between PM${}_{2.5}$ and influenza-like illness counts in the flu season.

Among the above four air pollutants, PM${}_{2.5}$ has received the most widespread attention in recent years. This may be due to the fact that PM${}_{2.5}$ is small enough to penetrate deep into the lungs, travels long distances and transcends boundaries or regions, and largely contributes to the impairment of visibility [27]. However, ignoring the impacts of other pollutants could lead to increasing health risks and detrimental climate changes in the long run. From the perspective of the tourism industry, overlooking the impact of other pollutants could lower travelers’ satisfaction with respect to tourist destinations and expose travelers to more serious health threats.

### 2.2. The Impact of Air Pollution on Tourism

Two major streams of studies have examined the impacts of air pollution on tourism. One stream of literature relied on questionnaire survey tools to measure travelers’ subjectively perceived level of air pollution, which is actually a psychological response to the actual air quality (e.g., [2,5,28,29]). Another stream of literature examined the impacts of actual air pollution on the tourism industry by applying different scientifically measured indices of air pollution (e.g., [9,10,11,30]). Table 1 presents a non-exhaustive summary of previous studies. The table reports the area studied, the period covered, and the type of pollutants studied by each research. As shown in the table, the air pollution indicators that were utilized to measure air quality varied across the different studies. It was found that PM${}_{2.5}$ and PM${}_{10}$ were two of the most frequently used air pollution indicators, followed by the comprehensive index of AQI, or the air pollution index (API). It was noticed that other air pollutants, such as SO${}_{2}$ and NO${}_{2}$, have been less focused on. Some studies also relied on the number of good or bad air-quality days within one year or the subjectively perceived level of air pollution reported in questionnaire surveys to measure air quality.

Overall, there was no consensus on the selection of air pollution indicators in the literature. The choice of air pollution indicator largely depended on the degree of convenience in data collection. Although different pollutants all reduce the quality of air, their respective impacts on tourist activities may be different. Interestingly, Yan et al. [8] reported that PM${}_{2.5}$, PM${}_{10}$, SO${}_{2}$, and NO${}_{2}$ all depressed humans’ leisure-relevant behaviors, while Zhou et al. [43] reported that only PM${}_{10}$ had a statistically significant impact. As an extension of these two studies, our study also examined and compared the regression results for different pollutants. However, differently from Yan et al. [8] who used geotagged social media check-in data of “Weibo” covering 2015 and 2016, and Zhou et al. [43], who solely concentrated on one city in China (the city of Beijing), this study used a city-level sample, including 337 Chinese cities and covering the period between 2007 and 2016. Based on a wider sample, this study was able to examine the impact of air pollution on both inbound and domestic tourism more precisely from an aggregate perspective.

## 3. Empirical Model and Data

### 3.1. Model

The study was based on a city-level sample with panel data structure, consisting of both temporal and spatial dimensions. Following the previous studies investigating the pollution-tourism nexus (e.g., [12,31,32,39]), it was assumed that the impacts of air pollution and other explanatory variables on tourism could be captured by a linear econometric regression model. To be precise, in this study the following panel data econometric model was used:

(1) $$y_{it}=x_{it}\beta +s_{i}+u_{t}+\varepsilon_{it},$$

where $y_{it}$ is the dependent variable in city *i* during period *t*. $x_{it}$ refers to a vector of explanatory variables. $s_{i}$ is the section-fixed effect, and $u_{t}$ is the time-fixed effect. $\varepsilon_{it}$ is the error term. β is a vector of parameters to be estimated.

In this study, we investigated the impacts of air pollution on both domestic tourism and inbound tourism. Thus, we separately considered two dependent variables: $Arrivals^{domestic}$, the domestic tourist arrivals (in 10,000 person-times), and $Arrivals^{inbound}$, the inbound tourist arrivals (in 10,000 person-times). As usual, in the econometric regressions, we used the logarithmic values of these two variables to deal with the scaling problem. Accordingly, the variations of dependent variables are expressed as percentage changes.

Among the explanatory variables, the core variable of interest was the air pollutant indicator. In this study, we inspected four important air pollution indicators: PM${}_{2.5}$, PM${}_{10}$, SO${}_{2}$, and NO${}_{2}$. These variables of air pollutants are expressed by their degrees of concentration density (mg/m${}^{3}$) in ambient air.

A set of control variables was contained in the regressions: Scenic, Hotel, Road, GovSize, Population, and GDPpc. (i) The first control variable, Scenic, measures the abundance of local tourism endowment. It was calculated by the logarithmic value of the number of 4A- and 5A-rated scenic spots within each city. Since a 5A-rated scenic spot is typically considered as much more attractive than a 4A spot [9,30], we assumed that one 5A spot equalled three 4A spots. To avoid the problem of logarithmic computation when a city has zero 4A and 5A spots, we assigned a value of 0.01 to the number of scenic spots when it was actually zero. (ii) The second control variable, Hotel, measured the availability of tourism-specific infrastructure. We used the star-rated hotels to proxy this, since hotels are one of the most crucial tourism infrastructures. This variable was calculated by the ratio of the number of hotels divided by local population (in ten thousand). (iii) The third variable was Road, the length of road (km) per area (km${}^{2}$). This was an indicator of the transportation infrastructure. (iv) GovSize was the government size, measured by the ratio of local government expenditure to GDP. This variable was used to capture the impact of the government on local tourism’s development. (v) Population was the logarithmic value of the local population (in ten thousand), as a control variable for the potential economies of scale in tourism development. (vi) The last control variable was GDPpc, the logarithmic value of real GDP per capita (RMB). The nominal GDP was deflated, taking 2000 as the base year. Since previous studies have found that tourism might have impacts on economic and social development, which are directly linked to the values of the control variables in the current period, we lagged these control variables for three periods to mitigate the potential endogeneity problem. The idea is that the three-period-lagged values of the control variables probably affect the current value of the dependent variable (through their impacts on the current values of the control variables), but the current dependent variable has no effect on the past value of the control variables. That way, the potential endogeneity caused by reverse causality from the dependent variable to explanatory variables was mitigated. Definitely, one limitation of using the lagged values of control variables is that the estimated coefficients of them may not accurately reflect the impacts of the variables in the current period. However, given the large benefit of using this approach to mitigate the endogeneity issue, its limitation was deemed acceptable, and hence, it has been widely used in applied economics research (e.g., [44,45]).

### 3.2. Data

The data of PM${}_{2.5}$ were collected from NASA’s Global Annual PM${}_{2.5}$ Grids data [46,47]. The data of PM${}_{10}$, SO${}_{2}$, and NO${}_{2}$ were mainly extracted from a series of yearly published environmental quality reports—“The Report on the State of the Environment of China” [48,49,50]. These reports were written by China’s Ministry of Environmental Protection (MEP), and later, by the Ministry of Ecology and Environment (MEE). These reports provided detailed official data of air quality in different areas of China since 2007. The reports did not offer city-level air pollution data for the years 2013 and 2015. We checked the China Statistical Yearbook on Environment and several province-level statistical yearbooks to supplement the missing data for some cities in 2013 and 2015, as well as some observations in other years. It is worth mentioning that the MEP, and later, the MEE, have also reported the PM${}_{2.5}$ data in recent years. However, the available sample size was much smaller compared to that based on NASA’s data. That is why we relied on the latter data source for PM${}_{2.5}$ in our empirical analysis. In fact, an examination of the overlapping sample of these two data sources would make it clear that they are both reliable and highly correlated, though the reported values are not directly comparable, due to the technological disparity in measurement. NASA’s PM${}_{2.5}$ data were constructed on the basis of the information supplied by the remote sensing measurements of satellites, whereas the data offered by the MEP and MEE were from the direct measurements in local observation stations. Although there were uncertainties associated with the remote sensing measurements (for example, affected by weather and the precision of the electrical instruments), the accuracy and reliability of the PM${}_{2.5}$ grids data have been highly appreciated. In fact, both data sources have been widely utilized in previous research (e.g., [8,9,11,12,42]). The data from the two data sources were highly correlated. For example, for the sample cities in the year of 2016, the Pearson correlation coefficient between PM${}_{2.5}$ values from the two data sources was 0.753, indicating a strong positive correlation.

The data of the dependent variables $Arrivals^{domestic}$ and $Arrivals^{inbound}$, and the control variables Hotel, Road, GovSize, Population, and GDPpc during the period 2007–2013 came from the China Statistical Yearbook for Regional Economy. For the period covered, this yearbook provided city-level data for almost all Chinese cities above the prefecture-level, though with occasional missing values. The data between 2014 and 2016 were obtained from the EPS database, available at its website: http://www.epschinadata.com. In addition, we checked different province-level statistical yearbooks or utilized the linear interpolation method to supplement some missing observations. The data of Scenic were collected from the public information released by the tourism-relevant local governmental sectors in different provinces.

Ultimately, our sample was comprised of unbalanced panel data covering 337 Chinese cities for the period between 2007 and 2016. This sample covered almost all regions in Mainland China, including all four province-level municipalities (Beijing, Tianjin, Shanghai, and Chongqing) and all prefecture-level administrative districts except Sansha City and Danzhou City of Hainan Province. Sansha and Danzhou, which were respectively established in 2012 and 2015, were excluded due to lack of statistical data. Table 2 shows the summary statistics for the variables used in empirical analyses. It is clear from the table that there were rich heterogeneities among the sample cities. The sample contained both less developed and well developed, small and large, and clean and severely polluted cities. Some cities had highly developed tourism industries, but the tourism size in some cities was quite small. Overall, our sample was highly representative and able to provide sufficient information on the general situation of China.

It is notable that the different air pollutants are probably correlated. Indeed, since human activities often emit more than one kind of pollutant, a district may be polluted by multiple pollutants simultaneously [51,52]. Moreover, since different pollutants may have complex chemical and physical interactions within the air, the degree of air pollution caused by one pollutant may be exacerbated by another one. Considering this, we had a concern that if the correlation among different pollutants was sufficiently high, there would be no way to distinguish different pollutants and use traditional econometric regressions to estimate their individual impacts on tourism. Table 3 shows the Pearson correlation coefficients among the four pollutants. From the table, we see that different pollutants are indeed positively correlated, as expected. However, the correlation coefficients are not very high and do not exceed 0.5. Thus, the indices of these four pollutants reflect different aspects of air pollution, and can be considered separately as different explanatory variables in the regression model.

## 4. Results

The regression results for Equation (1) are reported in this section. Section 4.1 discusses the estimated impacts of air pollutants on domestic tourism. Section 4.2 discusses the circumstances regarding inbound tourism.

### 4.1. Impacts of Air Pollutants on Domestic Tourism

Table 4 shows the estimated influences of different air pollutants on domestic tourism. First, we focused on PM${}_{2.5}$. As reported in column (1) of the table, the coefficient of PM${}_{2.5}$ was −4.815, statistically significant at the 1% level. This implies that if the PM${}_{2.5}$ density increased by 1 μg/m${}^{3}$ (= 0.001 mg/m${}^{3}$), domestic tourist arrivals would decline by 0.482%. Given that the mean value of annual domestic tourist arrivals among our sample cities was around 17 million person-times, this magnitude corresponds to a decline of 81,855 person-times in tourist arrivals. This is indeed a huge loss. Regarding the control variables, we found that the coefficients of Scenic and Hotel were both significantly positive, consistent with the straightforward idea that more scenic spots and more tourism infrastructure benefit tourism. Government size, GovSize, had a significant positive coefficient, perhaps because local government plays an important role in tourism development in China. The coefficient of GDP per capita, GDPpc, was also positive, indicating that, on average, Chinese tourists considered more developed regions to be more attractive. The variables Road and Population did not show significant impacts on domestic tourism.

To investigate the robustness of our finding on the harmful effect of PM${}_{2.5}$, we conducted three further robustness analyses on the result. (i) One concern is that air pollution and tourism might have complex reciprocal interactions [53], which might cause the endogeneity problem in the econometric estimation [9]. System GMM (general method of moments) estimation is a reliable approach to deal with the endogeneity problem in a “short panel” with many individuals but a small number of periods like our data structure. Column (2) of the table reports the result of System GMM estimation, which shows a significant negative coefficient of −2.136. The magnitude was smaller than that of the coefficient in column (1), but was still quite considerable. (ii) Comparing the number of observations of PM${}_{2.5}$ and the other three pollutants, as previously reported in Table 2, we found that PM${}_{2.5}$ data had more observations than the other three pollutants. This raised the concern that the regression results regarding PM${}_{2.5}$ might not be fully comparative to those for the other pollutants, due to the difference in sample size. To address this concern, we deleted the sample points that had data for PM${}_{2.5}$ but not for the other pollutants, and repeated the regression based on the smaller sample obtained. The estimated coefficient of PM${}_{2.5}$ was −5.304, as displayed in column (3). Clearly, our previous finding held. (iii) To date, we have only considered the impact of PM${}_{2.5}$ on tourist arrivals. In column (4), we report the estimate when the dependent variable was the logarithmic value of tourism receipts (in 100 million RMB, deflated based on the year 2000 price), instead of tourist arrivals. The estimated coefficient was −4.394, very close to that in column (1). Given that the mean annual domestic tourism receipt was 12 billion RMB, the coefficient implies that, on average, a 1 μg/m${}^{3}$ increase in PM${}_{2.5}$ concentration would cause a reduction of 53 million RMB (approximately 8 million US dollars) in domestic tourism receipts at the city level. In a nutshell, combining the results in columns (1)–(4) together, we are able to claim that PM${}_{2.5}$ had a robust and significant negative impact on domestic tourism.

Next, we examined the effects of PM${}_{10}$, SO${}_{2}$, and NO${}_{2}$ on domestic tourism, respectively. As reported in column (5), the estimation did not detect a statistically significant impact from PM${}_{10}$. Column (6) reports the estimated coefficient of SO${}_{2}$, which was not significant either. Similarly, as can be seen from column (7), NO${}_{2}$ did not significantly affect domestic tourism.

Lastly, we put all four pollutants into one regression equation and reported the estimates in column (8). The result still showed a significant negative coefficient for PM${}_{2.5}$, but not for PM${}_{10}$, SO${}_{2}$, or NO${}_{2}$. This result supported the findings from columns (1)–(7) when we checked the impacts of the four pollutants one by one.

### 4.2. Impacts of Air Pollutants on Inbound Tourism

Table 5 demonstrates the impacts of air pollutants on inbound tourism. Column (1) reports the baseline estimates for PM${}_{2.5}$. The statistically significant coefficient was −12.269, indicating that inbound tourist arrivals would decline by 1.227% in response to a 1 μg/m${}^{3}$ (=0.001 mg/m${}^{3}$) increase in PM${}_{2.5}$ concentration. Given that the mean value of annual inbound tourist arrivals in our sample cities was nearly 1 million person-times, this magnitude indicates a decline of 12,269 person-times in tourist arrivals. This loss is indeed substantial. The control variables were generally not statistically significant, indicating that inbound tourists were not sensitive to the economic and social characteristics of destination cities.

Three robustness analyses on the impact of PM${}_{2.5}$ are reported in columns (2)–(4). (i) In column (2), the System GMM estimates are reported. The coefficient of PM${}_{2.5}$ was −12.517, very close to that reported in column (1). (ii) In column (3), we relied on a smaller sample, in which all sample points had data for all four pollutants. The estimated coefficient of PM${}_{2.5}$ was −7.185. This coefficient was still significantly negative, supporting the result in column (1). (iii) In column (4), we used the logarithmic value of inbound tourism receipts (in 100 million RMB, deflated based on the year 2000 price) as the dependent variable, instead of tourist arrivals. The estimated significant negative coefficient of −8.259 supported the finding that PM${}_{2.5}$ harmed inbound tourism. The magnitude implies that inbound tourism receipts would decline by 7 million RMB (approximately 1 million US dollars) after PM${}_{2.5}$ concentration increased by 1 μg/m${}^{3}$, given that the average inbound tourism receipt of the sample cities was 893 million RMB per year.

Next, we checked the impacts of the other three air pollutant indices. The impacts of PM${}_{10}$ and SO${}_{2}$ were not significant, as reported in columns (5) and (6), respectively. From column (7), it was found that the impact of NO${}_{2}$ was negative and statistically significant at the 10% level. This implies that inbound tourists were responsive to the rise of NO${}_{2}$ pollution.

In column (8), we report the estimates after we put all four pollutants together within one regression equation. The coefficient of PM${}_{2.5}$ was −7.359 and maintained statistical significance. PM${}_{10}$ and SO${}_{2}$ did not have significant impacts. The coefficient of NO${}_{2}$ was significantly negative, analogous to that in column (7).

## 5. Discussion and Implications

### 5.1. Discussion

The analyses in this study provide three important findings. Firstly, it was found that air pollution, measured by PM${}_{2.5}$, shows a harmful effect on both domestic and inbound tourism. This finding is consistent with previous studies (e.g., [29,30,31,33]) that reported the negative impact of air pollution on tourism. As our sample covered a wide geographic area and a long time-span, this study supplements the prior literature by providing further evidence on the pollution-tourism nexus. As claimed in the previous studies, policy makers should take actions to mitigate the air pollution problem for the purpose of boosting tourism. Good air quality is a substantially attractive characteristic for tourist destination cities.

Secondly, different pollutants were found to exert different impacts on the tourism industry. According to our estimates, the most adverse pollutant indicator is PM${}_{2.5}$, which was compared to PM${}_{10}$, SO${}_{2}$, and NO${}_{2}$. The estimates demonstrate a robust, large, and statistically significant impact of PM${}_{2.5}$ on tourism. Given that PM${}_{2.5}$ can be especially harmful, due to its relatively small size compared to other air pollutants, it has attracted more public attention through microblogging platforms such as Weibo [54]. In addition, PM${}_{2.5}$ is more closely associated with the reduction of visibility than some other pollutants [55,56]. Travelers are highly concerned about the low visibility issue, as it can reduce the aesthetics of tourist attractions [5,57] and interrupt traffic by causing flight delays or cancellations, or highway closures [58]. Regarding the other three pollutants, PM${}_{10}$, SO${}_{2}$, and NO${}_{2}$, the estimation results show that they do not have a similar impact to that of PM${}_{2.5}$. No statistically significant effect of PM${}_{10}$ and SO${}_{2}$ on tourism was detected. NO${}_{2}$ was found to negatively influence inbound tourism, but it does not significantly affect domestic tourism. This finding is novel and not consistent with some previous studies, including Yan et al. [8], Yoon [41], and Zhou et al. [43], which reported a negative effect of PM${}_{10}$, SO${}_{2}$, or NO${}_{2}$ on tourism. The different impacts of NO${}_{2}$ on domestic and inbound tourism are especially interesting. There may be at least two plausible explanations. The first reason is relevant to the degree of perception and concern about air pollution in different tourist groups. The previous studies have confirmed that people’s opinions about the severity of air pollution largely depend on their sociodemographic status, including education, knowledge, income, and so on [59,60]. For instance, tourists with higher income levels are typically more sensitive to air pollution than those with low income [39]. It is possible that, on average, the sociodemographic characteristics of inbound tourists make them more aware of the damage of NO${}_{2}$, compared to domestic tourists in China. The second reason is relevant to the differences in the health risks faced by inbound and domestic tourists during the tourist activities. As the stay time of foreign tourists is usually longer than that of domestic tourists, inbound tourists are potentially exposed to more NO${}_{2}$ when they visit polluted cities. Therefore, inbound tourists might become more responsive to the variations of pollution. For example, Song et al. [61] demonstrated increasing prevalence trends of adult asthma in Asian regions, especially in Japan and South Korea, which are the top source countries of China’s inbound tourism. Given that exposure to NO${}_{2}$ could lead to asthma exacerbations [62], it is possible that people with potential asthma or other respiratory diseases would stay away from travel destinations with high NO${}_{2}$ concentrations.

It is notable that, although our study did not report as significant a harmful impact of PM${}_{10}$, SO${}_{2}$, or NO${}_{2}$ on tourism as PM${}_{2.5}$, this does not necessarily mean that these three pollutants are trivial to sustainable tourism development in China. From the perspective of public health, the threats to tourists’ health conditions posed by PM${}_{10}$, SO${}_{2}$, and NO${}_{2}$ should be noticed. It is also notable that, since the inference from our regressions reflects an average situation based on a sample of 337 Chinese cities, it does not rule out idiosyncratic properties in different areas. It is possible that, although PM${}_{2.5}$ is the most adverse pollutant on average, pollution problems in certain regions are majorly caused by other pollutants. The unequal impacts of different pollutants on tourism detected by our study essentially indicate that tourism-relevant policy makers and researchers should pay attention to monitoring suitable air pollution indictors. In particular, PM${}_{2.5}$ should not be ignored in tourism analysis.

The third finding was that domestic tourists and inbound tourists respond to air pollution at different magnitudes. According to our estimates, if PM${}_{2.5}$ concentration rises by 1 μg/m${}^{3}$, domestic and inbound tourist arrivals will decline by 0.482% and 1.227%, respectively. Thus, in terms of percentage change, inbound tourists are more sensitive to the degradation of air quality. It is plausible that foreign travelers are more aware of the harmfulness of air pollution, compared to Chinese travelers. An earlier study by Law and Cheung [33] has signaled that travelers from Western countries were more sensitive to the air pollution in Hong Kong than Asian travelers. Our study extends the result of Law and Cheung [33], which used Hong Kong as a case study, to a large geographic scope. In addition, it should be noticed that, as the aggregate size of domestic tourism is much larger than inbound tourism in China, in absolute values, the impact of air pollution on domestic tourism is much stronger. Our estimates imply a reduction of 81,855 person-times in annual domestic tourist arrivals and 12,269 in inbound tourist arrivals, in response to a 1 μg/m${}^{3}$ increase in PM${}_{2.5}$. These estimates could help the tourism sectors predict the trends and variations of domestic and inbound tourism development associated with varying air quality problems. Moreover, these estimates could not only exert pressure on policy makers to improve environmental outcomes, but also raise Chinese citizens’ awareness of environmental protection to build a positive destination image.

### 5.2. Implications

From a theoretical perspective, this study made the following contributions. First, this study empirically examined the impacts of four important components of air pollution (PM${}_{2.5}$, PM${}_{10}$, SO${}_{2}$, and NO${}_{2}$) on both the domestic and inbound tourism industries in China using a sample of 337 cities covering the period between 2007 and 2016. The sample used in the study may generate more precise and updated estimates, since it covers the period of recent years for a wide geographic range. Second, the findings enrich the air pollution–tourism nexus literature by confirming the finding from previous research that PM${}_{2.5}$ plays a vital role in depressing both domestic and inbound tourist numbers in China, and by providing new insights into how NO${}_{2}$ exerts different effects on the domestic and inbound tourism industries. The study results remind researchers that air pollution might be more accurately studied from the perspective of different air pollutants.

Practically, the results indicate that the Chinese government should continue tackling air pollution in China for the benefit of human health and for the sustainable development of the tourism industry. On the one hand, among the four common air pollutants considered, it seems that PM${}_{2.5}$ has received the most attention from travelers over the last decade. Therefore, tourism policy makers should primarily focus on PM${}_{2.5}$, concerning the nexus between air quality and the development of tourism. On the other hand, given the fact that other air pollutants could also result in negative health effects, emphasizing the importance of PM${}_{2.5}$ should not overshadow the threats posed by other air pollutants. It is suggested that great efforts should be made to raise travelers’ awareness of other air pollutants. Furthermore, although China’s outbound tourism market has attracted the attention of the world, its inbound tourism has been experiencing very slow growth [63]. As suggested by this study, NO${}_{2}$ pollution should also be tackled to attract more international travelers. The estimation results also reveal that inbound tourists are more sensitive to the air pollution issue in China. Given that Beijing, the capital of China with notorious air quality records, has attracted a lot of international attention in recent years, inbound tourists may believe that the air quality in other Chinese cities is also poor. In fact, there are a number of Chinese tourist cities with air quality up to standard, including Haikou, Zhoushan, Lhasa, Fuzhou, Zhuhai, and Huizhou, among others [64]. Destination marketers in China could strive to promote these cities to potential inbound tourists and design more haze-avoidance or smog-free travel packages.

## 6. Conclusions and Directions for Future Research

To conclude, the present study utilized an econometric model to empirically investigate how four atmospheric pollutants (PM${}_{2.5}$, PM${}_{10}$, SO${}_{2}$, and NO${}_{2}$) affected the tourism industry in China. The results of the analyses demonstrated that PM${}_{2.5}$ played a dominant role in negatively influencing China’s inbound and domestic tourism industries. The results also revealed that NO${}_{2}$ reduced the number of inbound tourists.

This study was restricted by several limitations, which actually indicate promising directions for future research. Firstly, some other air pollutants, such as CO (carbon monoxide) and O${}_{3}$ (ozone), were not investigated in this study due to the limitation of data availability. These two pollutants are also monitored and reported by the environmental sectors of the government in China. Unfortunately, data are only available for a very small sample from our data sources. In the future, the impact of other air pollutants could also be inspected if more data can be collected.

Secondly, this study examined the effects of different air pollutants but did not consider any comprehensive air pollution indices, such as AQI. Estimating the impact of AQI on tourism and comparing it with the estimated impact of PM${}_{2.5}$ will provide more information for better decision making. However, in this study, we were not able to do this because of the data availability problem. Given that AQI was not directly available from our data sources, in order to infer the values of AQI, we need to know the values of different pollutants, including PM${}_{2.5}$, PM${}_{10}$, SO${}_{2}$, NO${}_{2}$, CO, and O${}_{3}$. On the one hand, as mentioned previously, there were no sufficient data of CO and O${}_{3}$. On the other hand, our PM${}_{2.5}$ data provided by NASA were constructed based on the remote sensing measurements of satellites. The data are not directly comparable to those provided by the MEP and MEE based on direct measurements in different local observation stations, though they are both reliable and highly correlated. Hence, we can investigate the correlation between AQI and tourism in the future, after more data are released.

Thirdly, this study inspected the actual level of air pollution measured by scientific instruments. It is notable that the objectively measured air pollution level might not be completely consistent with people’s perceived level of air pollution, since the perception of air pollution is subjective and affected by a lot of social and individual factors, such as education and mass media. Future studies could collect data on the perceived air pollution level by potential tourists and examine whether the study results using subjective data match the results in this study.

## Figures and Tables

**Table 1:** A non-exhaustive summary of previous studies about the impact of air pollution on tourism.

Literature	Area Studied	Period Covered	Type of Pollutants Studied						
			PM_2.5_	PM_10_	SO_2_	NO_2_	AQI (or API)	Other Objective Indicators	Perceived Pollution
Anaman and Looi [31]	Brunei Darussalam	1995M1–1999M9						dummy variable for haze-related pollution	
Becken et al. [2]	China	2014							*√*
Chen et al. [32]	Sun Moon Lake Scenic Area, Taiwan, China	2004M1–2011M12						days of bad air quality	
Deng et al. [30]	31 provinces in China	2001–2013						industrial waste gas emission	
Dong et al. [9]	274 cities in China	2009–2012		*√*					
Dong et al. [10]	337 cities in China	2004–2013	*√*						
Law and Cheung [33]	Hong Kong, China	2003							*√*
Li et al. [34]	Beijing, China	2014							*√*
Liu et al. [11]	17 provinces in China	2005–2015	*√*						
Peng and Xiao [35]	Beijing, China	2016							*√*
Poudyal et al. [3]	Great Smoky Mountain National Park, USA	1988M3–2009M12						visibility of air	
Qiao et al. [36]	China	2015							*√*
Sun et al. [12]	28 cities in China	1999–2015	*√*						
Tang et al. [37]	Beijing, China	2004M1–2015M12					*√*		
Wang and Wang [38]	35 OECD countries	1995–2014						$CO_{2}$ emission	
Wang et al. [39]	11 cities in China	2016M1D1–2016M12D31					*√*		
Xu and Reed [28]	China	2006–2014							*√*
Xu and Reed [29]	Shanghai, China	2011M12–2016M10	*√*						*√*
Xu et al. [40]	174 cities in China	1998–2016	*√*						
Yan et al. [8]	251 cities in China	2015M1D1–2016M10D30	*√*	*√*	*√*	*√*	*√*	CO	
Yoon [41]	Seoul, South Korea	2015M4–2017M2		*√*					
Zhang et al. [5]	Beijing, China	2014							*√*
Zhang et al. [4]	Thailand	2001–2017						$CO_{2}$ emission	
Zhou et al. [42]	24 cities in China	2007M1–2012M12					*√*		
Zhou et al. [43]	Beijing, China	2005–2016	*O*	*√*	*O*	*O*		days of good air quality	

**Note**: (1) The symbol “*√* ” indicates that the corresponding air pollutant indicator was used in the study and demonstrated a statistically significant impact on tourism. The symbol “*O*” indicates that the corresponding air pollutant indicator was used in the study but did not demonstrate statistical significance. (2) The sample period covered in each study is described in the column "Period Covered". For the study based on the questionnaire survey to measure the perceived degree of air pollution, rather than the objectively measured level of air pollution, the "Period Covered" refers to the time of conducting questionnaire survey.

**Table 2:** Summary statistics.

Variable		Unit	Obs	Mean	SD	Min	Max
Dependent Variable	$Arrivals^{domestic}$	$10^{4}$ person-times	2892	6.832	1.229	1.033	10.658
	$Arrivals^{inbound}$	$10^{4}$ person-times	2952	1.447	2.311	−9.210	7.106
Air Pollutant	$PM_{2.5}$	mg/m${}^{3}$	3364	0.033	0.018	0.002	0.087
	$PM_{10}$	mg/m${}^{3}$	2376	0.083	0.033	0	0.436
	$SO_{2}$	mg/m${}^{3}$	2379	0.033	0.018	0.002	0.148
	$NO_{2}$	mg/m${}^{3}$	2379	0.030	0.012	0.002	0.069
Control Variable	Scenic	-	3367	−0.334	2.640	−4.605	4.331
	Hotel	-	3367	0.143	0.226	0.003	4.338
	Road	km/km${}^{2}$	3367	0.767	0.497	0.003	2.249
	GovSize	-	3367	0.189	0.179	0.040	3.581
	Population	$10^{4}$ persons	3367	5.665	0.877	2.077	7.996
	GDPpc	RMB	3367	9.720	0.731	7.613	11.874

**Note**: (1) The variables Scenic, Hotel, and GovSize have no unit. Scenic is the number of scenic spots. Hotel is the ratio of the number of hotels divided by local population (in ten thousand). GovSize is the ratio of government spending to GDP. (2) The variables $Arrivals^{domestic}$, $Arrivals^{inbound}$, Scenic, Population, and GDPpc were log-transformed. (3) The abbreviations “Obs”, “SD”, “Min”, and “Max” in the first row denote “Observations”, “Standard Deviation”, “Minimum”, and “Maximum”, respectively.

**Table 3:** Correlation coefficients among the four pollutants.

	${\mathrm{PM}}_{2.5}$	${\mathrm{PM}}_{10}$	${\mathrm{SO}}_{2}$	${\mathrm{NO}}_{2}$
$PM_{2.5}$	1			
$PM_{10}$	0.329	1		
$SO_{2}$	0.309	0.403	1	
$NO_{2}$	0.435	0.492	0.401	1

**Table 4:** The impacts of air pollutants on domestic tourism.

Variable	PM${}_{2.5}$				PM${}_{10}$	SO${}_{2}$	NO${}_{2}$	All Pollutants
	Baseline	System GMM	Smaller Sample	Tourism Receipts				
	(1)	(2)	(3)	(4)	(5)	(6)	(7)	(8)
$PM_{2.5}$	−4.815 ***	−2.136 *	−5.304 ***	−4.394 **				−5.376 ***
$PM_{10}$					0.558			0.817
$SO_{2}$						−0.589		−0.768
$NO_{2}$							0.001	0.070
Scenic	0.016 ***	0.138 ***	0.011 **	0.024 ***	0.012 **	0.012 **	0.012 **	0.011 **
Hotel	0.176 ***	0.068	0.147 ***	0.055	0.145 ***	0.149 ***	0.147 ***	0.146 ***
Road	−0.006	0.275 **	0.091 *	−0.199 ***	0.111 **	0.111 **	0.113 **	0.084
GovSize	0.539 ***	−0.238	0.594 **	0.313	0.569 **	0.555 **	0.551 **	0.598**
Population	0.310	0.805 ***	0.238	0.014	0.267	0.280	0.282	0.220
GDPpc	0.192 **	0.048	0.227 **	0.298 ***	0.230 **	0.227 **	0.228 **	0.231 **
Observations	2892	2892	2033	2783	2033	2036	2036	2033
Cities	337	337	328	337	328	328	328	328
$R^{2}$	0.776	-	0.815	0.738	0.814	0.814	0.814	0.816

**Statistical significance**: * *p* < 10%, ** *p* < 5%, *** *p* < 1%.

**Table 5:** The impacts of air pollutants on inbound tourism.

Variable	PM${}_{2.5}$				PM${}_{10}$	SO${}_{2}$	NO${}_{2}$	All Pollutants
	Baseline	System GMM	Smaller Sample	Tourism Receipts				
	(1)	(2)	(3)	(4)	(5)	(6)	(7)	(8)
$PM_{2.5}$	−12.269 ***	−12.517 **	−7.185 *	−8.259 *				−7.359 *
$PM_{10}$					0.924			1.780
$SO_{2}$						−0.567		0.804
$NO_{2}$							−5.069 *	−6.625 *
Scenic	0.002	0.455 ***	0.011	0.046 ***	0.012	0.012	0.010	0.008
Hotel	0.105	2.034 *	0.085	0.089	0.080	0.086 *	0.094 *	0.086 *
Road	−0.182	−1.071 **	−0.283 **	−0.198	−0.262 *	−0.260 *	−0.239 *	−0.268 *
GovSize	−0.472 **	−11.859 ***	0.090	−0.656 **	0.064	−0.024	−0.021	0.094
Population	−0.246	−0.306	−0.667	−1.150	−0.623	−0.643	−0.684	−0.733
GDPpc	0.031	0.152	−0.052	0.034	−0.039	−0.053	−0.047	−0.033
Observations	2952	2952	2111	2941	2111	2114	2114	2111
Cities	337	337	324	337	324	324	324	324
$R^{2}$	0.214	-	0.266	0.100	0.265	0.262	0.264	0.270

**Statistical significance**: * *p* < 10%, ** *p* < 5%, *** *p* < 1%.
